# Supplementary material for: Rad53 regulates the lifetime of Rdh54 at homologous recombination intermediates
Source: Nucleic Acids Res. 2023 Oct 18;51(21):11688–705. doi: 10.1093/nar/gkad848 (PMC10681728; doi:10.1093/nar/gkad848)
Supplement: gkad848_Supplemental_File [file gkad848_supplemental_file.pdf]

Supplemental Information

Supplemental Table 1

Strains	Genotype	Source or reference
LSY-2202-15D (WT)	*MATa ade2-n his3::NatMX4 met22:klURA3*	*(Mazon et al. 2010)*
LSY-2205-11C (WT)	*MATalpha ade2-I lys2:GAL-ISCE1 his3:HphMX4*	*(Mazon et al. 2010)*
JBC0011 (15D)	*RDH54::RDH54-KanMX*	*(Keymakh et al. 2022)*
JBC0013 (11C)	*RDH54::RDH54-KanMX*	*(Keymakh et al. 2022)*
JBC0014 (15D)	*RDH54::rdh54K318R*	*(Keymakh et al. 2022)*
JBC0016 (11C)	*RDH54::rdh54K318R*	*(Keymakh et al. 2022)*
JBC0018 (15D)	*RDH54::rdh54T851A*	*This study*
JBC0020 (11C)	*RDH54::rdh54T851A*	*This study*
JBC0026 (15D)	*RDH54::rdh54S852A*	*This study*
JBC0028 (11C)	*RDH54::rdh54S852A*	*This study*
JBC0054 (15D)	*RDH54::rdh54S852D*	*This study*
JBC0060 (11C)	*RDH54::rdh54S852D*	*This study*
JBC0056 (15D)	*RDH54::rdh54T851D*	*This study*
JBC0062(11C)	*RDH54::rdh54T851D*	*This study*
JBC0086 (15D)	*RDH54::rdh54T851A,S852A*	*This study*
JBC0092 (11C)	*RDH54::rdh54T851A,S852A*	*This study*
JBC0080 (15D)	*RDH54::HIS3*	*This study*
JBC0083(11C)	*RDH54::HIS3*	*This study*
BY4741 (WT)		*Dharmacon*
BY4741	*rdh54Δ*	*Dharmacon*
HK663A-leu2-R1 (A)	*leu2-EcoRI RAD5+ ade2-1 can1-100 his3-11,15 trp1-1 ura3*	*(Klein H 1997)*
HK6793B-leu2BSTEII (alpha)	*leu2-BstEII RAD5+ ade2-1 can1-100 his3-11,15 trp1-1 ura3*	*(Klein H 1997)*
JBC206	*leu2-EcoRI RAD5+ ade2-1 can1-100 his3-11,15 trp1-1::TRP1 ura3*	*This study*
JBC207	*leu2-BstEII RAD5+ ade2-1 can1-100 his3-11,15 trp1-1 ura3::URA3*	*This study*
JBC0174	*JBC206 RDH54::RDH54-KanMX*	*This study*
JBC0175	*JBC206 RDH54::rdh54T851A-KanMX*	*This study*
JBC0176	*JBC206 RDH54::rdh54T851D-KanMX*	*This study*
JBC0177	*JBC206 RDH54::rdh54S852A-KanMX*	*This study*
JBC178	*JBC206 RDH54::S852D-KanMX*	*This study*

JBC179	*JBC206 RDH54::rdh54T851A,S852A-KanMX*	*This study*
JBC0180	*JBC207 RDH54::RDH54-KanMX*	*This study*
JBC0181	*JBC207 RDH54::rdh54T851A-KanMX*	*This study*
JBC0182	*JBC207 RDH54::rdh54T851D-KanMX*	*This study*
JBC0183	*JBC207 RDH54::rdh54S852A-KanMX*	*This study*
JBC0184	*JBC207 RDH54::S852D-KanMX*	*This study*
JBC0185	*JBC207 RDH54::rdh54T851A,S852A-KanMX*	*This study*
JBC228	*JBC206 RDH54::KanMX*	*This study*
JBC229	*JBC207 RDH54::KanMX*	*This study*
yRA53	*MAT*a::DEL Hocs::hisG uraD851trp1DEL63 leu2::KAN hmlDEL::hisGHMR::ADE3 ade3::GAL::Hocan1DEL::UR::Hocs::NAT, RA::LEU2, A3::TRP1	*Anand et al 2014*
yRA163	*yRA53 RDH54::HPHMX*	*Anand et al 2014*
JBC0084	*yRA53 leu2::KAN::HPHMX*	*This study*
JBC0099	*JBC0084 RDH54::RDH54KanMX*	*This study*
JBC0100	*JBC0084 RDH54::rdh54T851A*	*This study*
JBC0101	*JBC0084 RDH54::rdh54T851D*	*This study*
JBC0102	*JBC0084 RDH54::rdh54S852A*	*This study*
JBC0103	*JBC0084 RDH54::rdh54S852D*	*This study*
JBC0162	*JBC0084 RDH54::rdh54T851A,S852A*	*This study*
JKM179	*hoΔ hml::ADE1 MATα hmr::ADE1 ade1-110 leu2,3-112 lys5 trp1::hisG ura3-52 ade3::GAL:HO*	*Kind gift from J. Haber*
JBC0144	*JKM179 RDH54::RDH54-3xHA-KanMX*	*This study*
JBC0146	*JKM179 RDH54::rdh54T851A-3xHA-KanMX*	*This study*
JBC0148	*JKM179 RDH54::rdh54T851D-3xHA-KanMX*	*This study*
JBC0152	*JKM179 RDH54::rdh54S852A-3xHA-KanMX*	*This study*
JBC0252	*JKM179 RDH54::rdh54S852D-3xHA-KanMX*	*This study*
JBC0164	*JKM179 RDH54::rdh54T851A,S852A-3xHA-KanMX*	*This study*
Diploid (JBC0011 X JBC0013)	*RDH54::RDH54-KanMX/ RDH54::RDH54-KanMX*	*This study*
Diploid (JBC0011XJBC0016)	*RDH54::RDH54-KanMX /RDH54::rdh54K318R*	*This study*
Diploid (JBC0011XJBC0020)	*RDH54::RDH54-KanMX/ RDH54::rdh54T851A*	*This study*
Diploid (JBC0018XJBC0020)	*RDH54::rdh54T851A/ RDH54::rdh54T851A*	*This study*
Diploid (JBC0026X0028)	*RDH54::rdh54S852A/ RDH54::rdh54S852A*	*This study*
Diploid(JBC0054XJBC0060)	*RDH54::rdh54S852D/ RDH54::rdh54S852D*	*This study*
Diploid(JBC0056XJBC0062)	*RDH54::rdh54T851D/RDH54::rdh54T851D*	*This study*

Diploid (JBC0086XJBC0092)	*RDH54::rdh54T851A,S852A/RDH54::rdh54T851A,S852A*	*This study*
Diploid (JBC174XJBC180)	*RDH54::RDH54-KanMX/ RDH54::RDH54-KanMX*	*This study*
Diploid (JBC175XJBC181)	*RDH54::rdh54T851A/ RDH54::rdh54T851A*	*This study*
Diploid (JBC176XJBC182)	*RDH54::rdh54T851D/RDH54::rdh54T851D*	*This study*
Diploid (JBC177XJBC183)	*RDH54::rdh54S852A/ RDH54::rdh54S852A*	*This study*
Diploid (JBC178XJBC184)	*RDH54::rdh54S852D/ RDH54::rdh54S852D*	*This study*
Diploid (JBC179XJBC185)	*RDH54::rdh54T851A,S852A/RDH54::rdh54T851A,S852A*	*This study*
Diploid (JBC0080XJBC0083)	*RDH54::HIS3/RDH54::HIS3*	*This study*
Diploid (JBC228XJBC229)	*RDH54::KanMX/RDH54::KanMX*	*This study*

Supplemental Table 1: Yeast strains used in this study. These are the yeast strains used in this study.

Supplemental Table 2

**Plasmid backbone**	**Construct**	**Source**
pET15	6xHis-Rdh54-WT	**This study**
pET15	6xHis-Rdh54K318R	**This study**
pET15	6xHis-Rdh54T851A	**This study**
pET15	6XHis-Rdh54T851D	**This study**
pET15	6xHis-Rdh54S852A	**This study**
pET15	6xHis-Rdh54S852D	**This study**
pET15	6xHis-Rdh54T851AS852A	**This study**
pET15	6xHis-Rdh54-mCherry-WT	**This study**
pET15	6xHis-Rdh54-mCherryK318R	**This study**
pET15	6xHis-Rdh54-mCherryT851A	**This study**
pET15	6xHis-Rdh54-mCherryS852A	**This study**
pET15	6xHis-Rdh54-mCherryT851D	**This study**
pET15	6xHis-Rdh54-mCherryS852D	**This study**
pET15	6xHis-Rdh54-mCherryT851AS852A	**This study**
pRS415	*RDH54-WT*	**This study**
pRS415	*rdh54S21A*	**This study**
pRS415	*rdh54S167A*	**This study**
pRS415	*rdh54S387A*	**This study**
pRS415	*rdh54S621A*	**This study**
pRS415	*rdh54S790A*	**This study**
pRS415	*rdh54S791A*	**This study**
pRS415	*rdh54T851A*	**This study**
pRS415	*rdh54S852A*	**This study**
pRS415	*rdh54K318R*	**This study**

pRS415	*rdh54K318RT851A*	**This study**
pRS305	*RDH54-KanMX*	**This study**
pRS305	*rdh54T851A-KanMX*	**This study**
pRS305	*rdh54T851D-KanMX*	**This study**
pRS305	*rdh54S852A-KanMX*	**This study**
pRS305	*rdh54S852D-KanMX*	**This study**
pRS305	*rdh54K318R-KanMX*	**This study**
pRS305	*rdh54T851A,S852A-KanMX*	**This study**
pET15	6xHis-Rad53	**A kind gift from Dirk Remus**
pET15	6xHis-Rad53D339A	**A kind gift from Dirk Remus**
pET15	6xHis-GFP-Rad53	**This study**
pET15	6xHis-GFP-Rad53D339A	**This study**

Supplemental Table 2: Plasmids used in this study. Plasmids are available upon request.

Supplemental Table 3

Strain	Selective Media	Leu+ Frequency (Total Population)
WT	345	$1.00x10^{-5}$
rdh54T851A:rdh54T851A	42	$0.45x10^{-5}$
rdh54T851D:rdh54T851D	54	$1.25x10^{-5}$
rdh54S852A:rdh54S852A	51	$0.58x10^{-5}$
rdh54S852D:rdh54S852D	243	$0.77x10^{-5}$
rdh54T851A,S852A:rdh54T851A,S852A	68	$0.77x10^{-5}$
rdh54:rdh54	258	$1.00x10^{-5}$

Supplemental Table 3: Table of Leu+ frequency for each of the yeast strains tested in this study. The number represent the total number of colonies counted on selective media and their frequency in the population.

Supplemental Table 4

Strain	CO	NCO	BIR	Total	Chromosome Loss
WT	94	72	8	174	0
rdh54T851A:rdh54T851A	76	611	17	704	0
rdh54T851D:rdh54T851D	122	189	32	343	0
rdh54S852A:rdh54S852A	42	93	25	160	0
rdh54S852D:rdh54S852D	74	343	31	448	1
RDH54:rdh54T851A	64	125	7	196	0
RDH54:rdh54K318R	62	125	9	183	0
rdh54T851A,S852A:rdh54T851A,S852A	186	181	12	379	0

Supplemental Table 4: The sum of the homologous recombination types identified in red/white assay for all mutant strains tested.

Supplemental Table 5

		In vitro			
In vivo SuperPhos	Both	WT-Rad53	Rad53D339A	Rdh54 No Rad53 (-ATP)	Rdh54 No Rad53 (+ATP)
S19 (1)	S19	S19	S673	S158	S158
S51		T23			
S165		T28			
S386		T30			
S619		S48			
S790		T54			
S791		S169			
T851 (3)		S189			
S852 (57)		S194			
S901 (1)		T197			
		S456			
		S587			
		T637			
		T691			
		S692			
		S705			
		S738			
		S775			
		S846			
		T847			
		T849			
	T851	T851			
	S852	S852			
		S872			
	S901	S901			

Phosphorylated residues identified by mass spectrometry

Supplemental Table 5: Residues that were identified as phosphorylated after in vitro phosphorylation under several different conditions. Also included are residues that are identified from the SuperPhos database.

Supplemental Figure 1

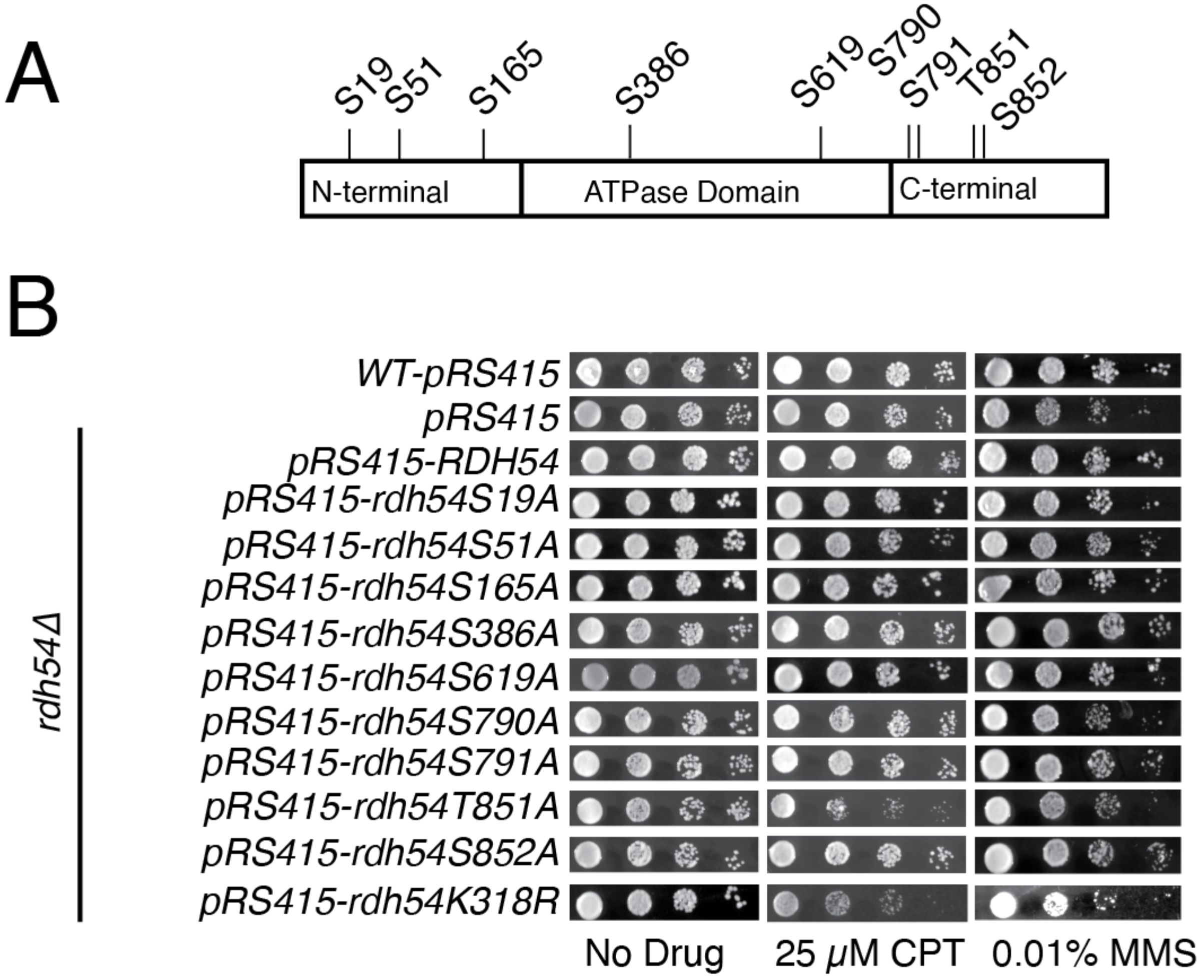

Supplemental Figure 1: *rdh54T851A* shows enhanced DNA damage phenotype
(A). Schematic diagram illustrating Rdh54 phosphorylation site as identified by the database SuperPhos. All S/T were converted to A. (**B).** Yeast growth assays to measure the growth of different *RDH54* alleles in the presence of 25 μM CPT or 0.01% MMS.

Supplemental Figure 2

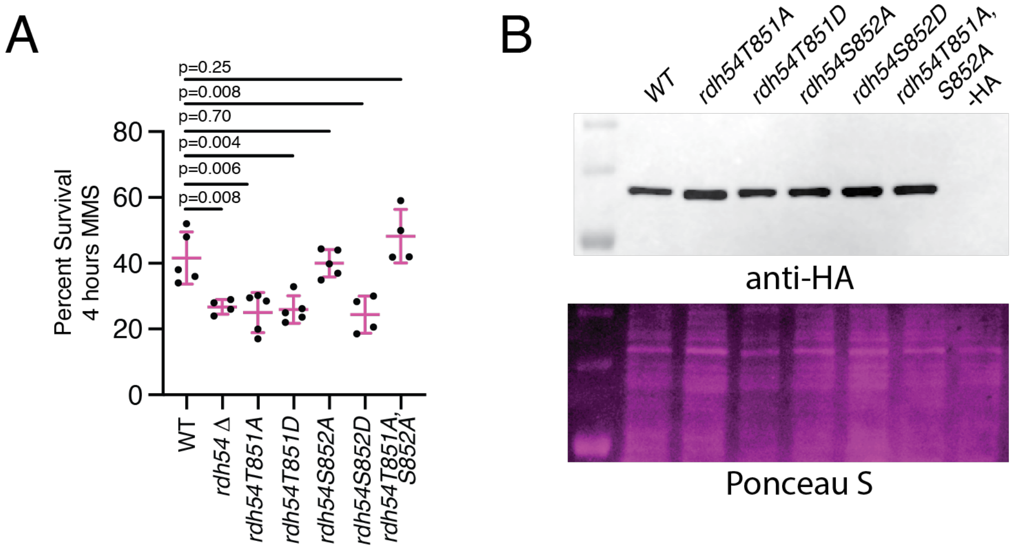

Supplemental Figure 2: Rdh54 mutants present MMS sensitivity
(A). Graph representing the percent survival of strains treated with 0.01% MMS for 4 hours. The Strains are WT (W303), *rdh54*Δ, *rdh54T851A*, *rdh54T851D*, *rdh54S852A*, *rdh54S852D*, and *rdh54T851A,S852A.* The bars represent the mean of the data, and the error bars represent the standard deviation of at least four independent experiments. P-values were generated using the student's t-test. (B). Western blot of HA tagged versions of RDH54, *rdh54T851A*, *rdh54T851D*, *rdh54S852A*, *rdh54S852D*, and *rdh54T851A,S852A* that illustrates comparable expression.

Supplemental Figure 3

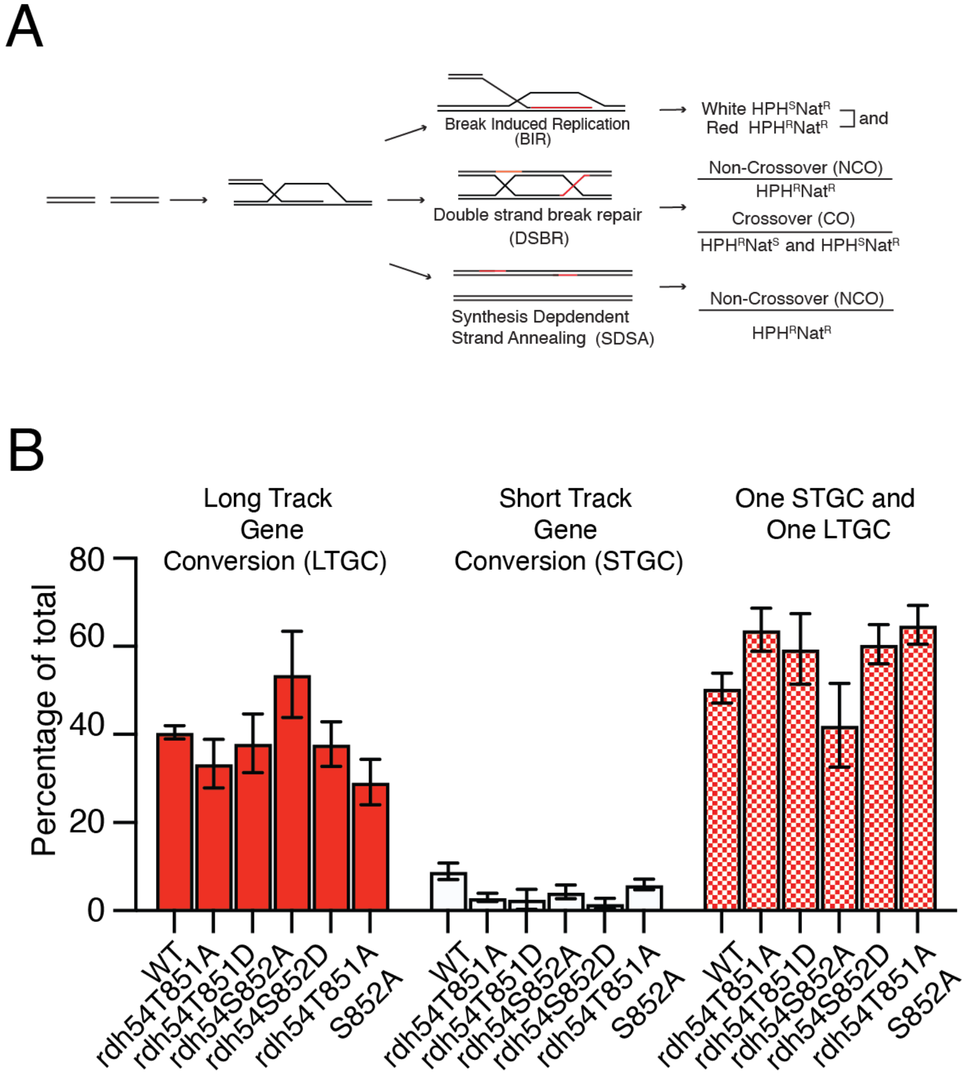

Supplemental Figure 3: Rdh54 mutants do not affect gene conversion

(A). Schematic outcome depicting the HR outcomes that can be deduced from the red/white assay. These outcomes result in different levels of genetic exchange between chromosomes and are inferred from color and antibiotic sensitivity of specific sectors. **(B).** Bar graph illustrating the populations of solid red colonies (LTGC), solid white colonies (STGC), and mixed sectored colonies (One STGC/ One LTGC). The bars represent the mean, and the error bars represent the standard deviation of at least three independent experiments.

Supplemental Figure 4

A

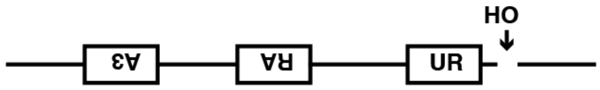

B

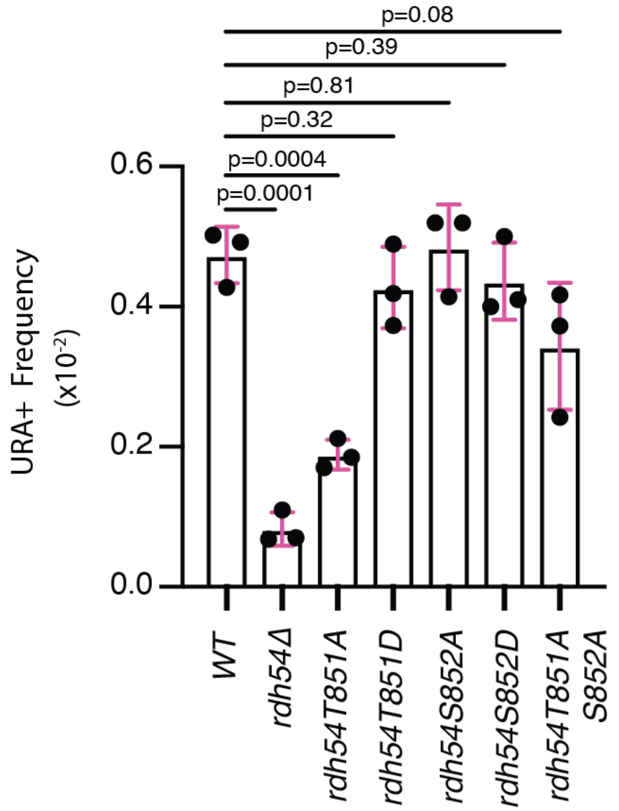

Supplemental Figure 4: *RDH54* mutants are also defective in DNA template switching
(A). Template defining assay used to measure intrachromosomal template switching during DSBR. The completion of an intact *URA3* gene requires a template switch to occur during repair. **(B).** Measured Ura+ frequency for *RDH54* WT and different alleles of *RDH54*. The bar represents the mean, and the error bars represent the standard deviation for three independent experiments. The p-values were generated by a two tailed t-test.

Supplemental Figure 5

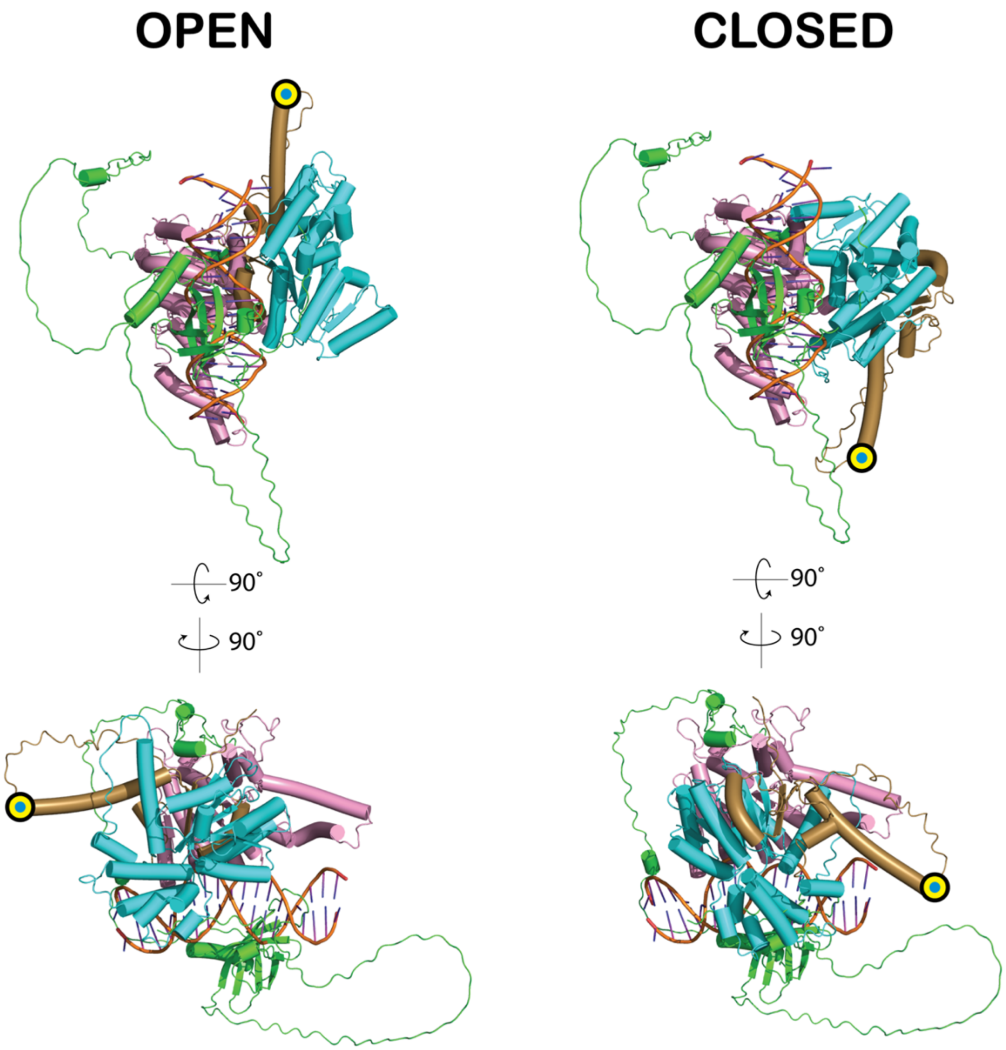

Supplemental Figure 5: Additional views of the open and closed models of yRdh54
To generate the open and closed models of yRdh54, the two RecA-like domains in the AlphaFold predicted structure of yRdh54 (https://www.uniprot.org/uniprotkb/B3LN76) were individually superimposed onto the equivalent domains of either the *Sulfalobus solfataricus* SWI2/SNF2 ATPase in the DNA-bound open state (PDB:1Z63), or AMP-PNP:DNA bound PcrA (PDB:3PJR) in the closed state. Most of the N-terminal domain of yRdh54 (green, residues: 1-261) is predicted to be unstructured (per-residue confidence (pLDDT) score < 50) and its position relative to other parts of the model cannot be interpreted. Lobe 1 (residues: 262-259), pink; lobe 2 (residues: 550-807), cyan; C-terminal domain (residues: 808-924), brown. The position of T851 is indicated with a yellow and cyan bullseye. DNA coordinates (orange) are from the *S. solfataricus* ATPase X-ray crystal structure (PDB:1Z63).

Supplemental Figure 6

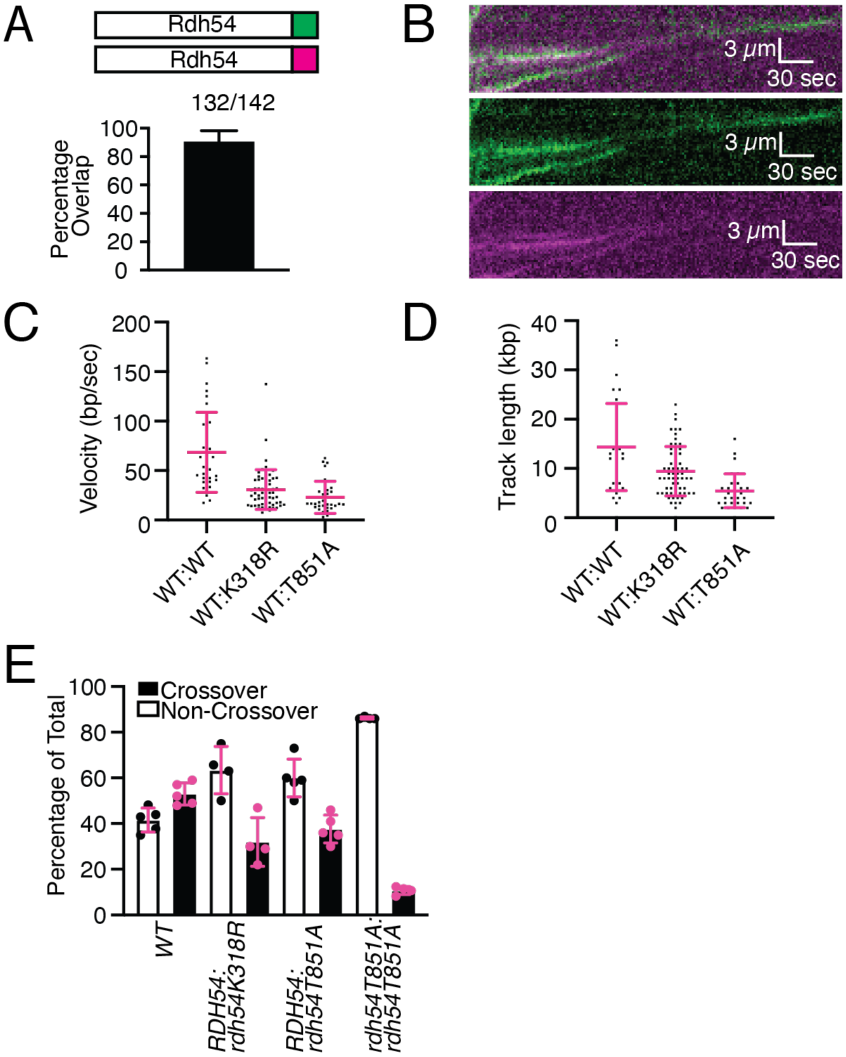

Supplemental Figure 6: Rdh54 forms a mixed oligomer during translocation
(A). Schematic illustrating experiments performed with equal molar Rdh54-mCherry and Rdh54-GFP (Top). Graphical representation of the number of Rdh54-mCherry molecules that overlap with Rdh54-GFP molecules. The error bar represents the standard deviation of three independent experiments (Bottom). **(B).** Representative kymographs illustrating the co-translocation of Rdh54-mCherry and Rdh54-GFP molecules. The channels are merged (top), magenta (middle), and green (bottom). **(C).** Dot plot representing the velocities (bp/sec) of mixed WT:WT (1:1) (N=32), WT:Rdh54K318R (1:1) (N=62), and WT:Rdh54T851A (1:1) (N=31). The bar and the error bars represent the mean and standard deviation of the data. **(D).** Dot plot of the track lengths (kbp) of mixed WT:WT (1:1) (N=32), WT:Rdh54K318R (1:1) (N=62), and WT:Rdh54T851A (1:1) (N=31). The bar and the error bars represent the mean and standard deviation of the data. **(E).** Percentage of CO and NCO outcomes for WT, *RDH54:rdh54K318R*, and *RDH54:rdh54T851A* strains. The bar represents the mean, and the error bars represent the standard deviation of four independent experiments from four different zygotes.

Supplemental Figure 7

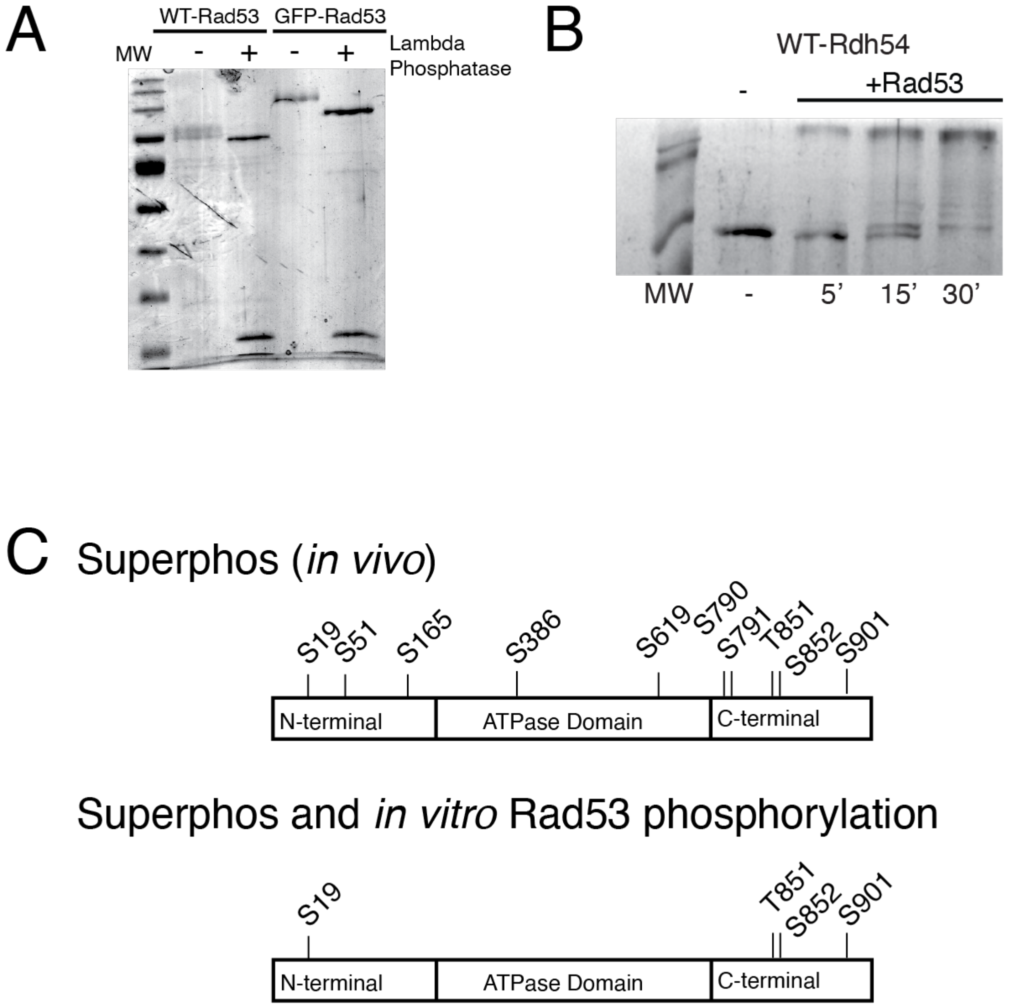

Supplemental Figure 7: Rdh54 directly interacts with and is a target of Rad53
(A). Representative SDS-PAGE illustrating purified Rad53 and GFP-Rad53 with and without Lambda phosphatase illustrating that Rad53 and GFP-Rad53 are purified in their phosphorylated form. **(B).** SDS-PAGE phostag gel with un-phosphorylated Rdh54, and Rdh54 phosphorylated by Rad53 for 5’, 15’, and 30’. **(C).** Schematic diagram of phosphorylation sites identified in the SuperPhos database (Top, reproduced from Supplemental Figure 1 and Supplemental Table 5) and phosphorylation sites identified both from SuperPhos and from an *in vitro* kinase reaction with Rad53 (Bottom, the data can also be found in Supplemental Table 5).

Supplemental Figure 8

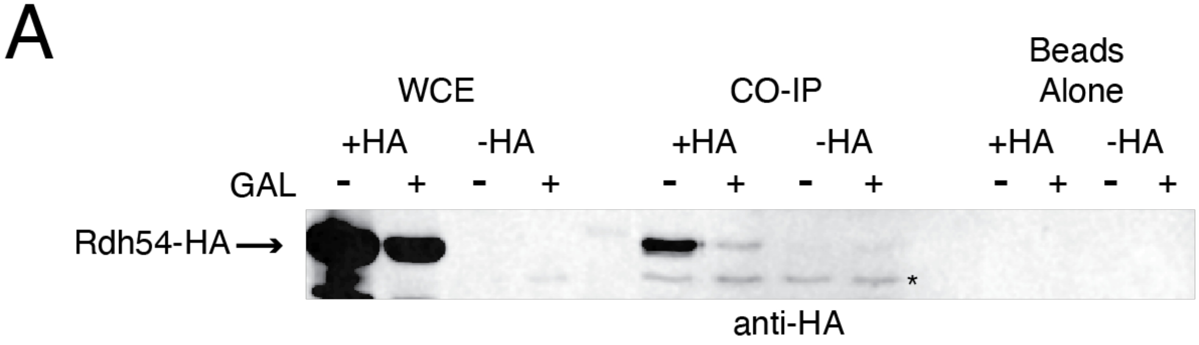

Supplemental Figure 8: Rdh54 co-immunoprecipitated with Rad53
(A). Representative western blot illustrating the CO-IP of Rdh54-HA with Rad53. Samples shown are from strains with an HA tagged Rdh54 and NO HA tag on the Rdh54. For WCE, IP'd sample and Beads alone. * is an unspecific band.
